# Treatise on Diagnostic Methods

**Published:** 1911-05

**Authors:** 


**Treatise on Diagnostic Methods.** By Professor **Dr. Herman Sahli,** Director of the Medical Clinic, University of Bern. Edited by **Nathaniel Bowditch Potter,** M.D., Assistant Professor of Clinical Medicine at Columbia University, New York. Second edition. Octavo, pp. 1229. Illustrated. Philadelphia and London: W. B. Saunders Co. 1911, (Cloth, $6.50.)

The second edition of this popular work follows closely the lines laid down by the distinguished Swiss clinican in his masterly revision. The entire field has been exhaustively reviewed and every diagnostic aid of importance has been noticed. The chief subjects where changes from the first issue appear are: The chapter on icterus has been recast and rewritten emphasizing Geraudel's investigations which have opened up new avenues of thought in the pathology of this condition.

The section on edema has been thoroughly revised; a brief section on the etiology of the various forms of dyspnea has been introduced—numerous additions and improvements have been made to the section on urine examinations. Tests for new substances have been included in the section on the detection of drugs and poisons in the urine. The chapter on the examination of the intestines has been much enlarged. Modern rectoscopy has led to such an advance in the early diagnosis of high rectal carcinoma that it should be completely mastered by the average physician; in the chapter on rhinoscopy a number of cuts of rhinoscopic findings in empyema of the accessory sinuses are of great practical value. J. A. R.

